# Recurrence of Thrombotic Thrombocytopenic Purpura after mRNA-1273 COVID-19 Vaccine Administered Shortly after COVID-19

**DOI:** 10.1155/2021/4130138

**Published:** 2021-09-13

**Authors:** Korin Karabulut, Ana Andronikashvili, Ahmet Hakki Kapici

**Affiliations:** ^1^Steward Carney Hospital, 2100 Dorchester Avenue, Boston, MA 02124, USA; ^2^Robert Wood Johnson University Hospital, 1 Robert Wood Johnson Place, New Brunswick, NJ 08901, USA

## Abstract

Thrombotic thrombocytopenic purpura (TTP) is a potentially life-threatening consumptive coagulopathy requiring emergent diagnosis and timely treatment. It is characterized by microangiopathic hemolytic anemia and thrombocytopenia with the development of microthrombi caused by inherited or acquired deficiency of the von Willebrand factor-cleaving protease ADAMTS13 and resulting end-organ damage. Most of the cases are the result of acquired deficiency of ADAMTS13, for which the exact etiology is unknown but reported to be related to various autoimmune disorders, infections, and medications. Our case report features of a patient with a history of idiopathic thrombocytopenic purpura and thrombotic thrombocytopenic purpura, who developed a recurrence of TTP 5 days after his first dose of the mRNA Coronavirus disease 2019 (COVID-19) vaccine (mRNA-1273 vaccine) in the setting of recent COVID-19. The close temporal association between vaccine administration, recent COVID-19, and relapse of remitted TTP raises concern for an enhanced immune reaction to COVID-19 vaccine in the setting of recent COVID-19 and underlying autoimmune disease. The association is not absolute, but given the novelty of COVID-19 and the mRNA COVID-19 vaccine and the relapse timing, it leads us to pose this hypothesis. Vaccine distribution to a larger and more diverse population will allow for an increased rate of adverse event reporting. This case report exemplifies potential safety issues that may be encountered with new vaccine administration in patients with recent COVID-19 and underlying autoimmune disease. There are no specific recommendations for COVID-19 vaccine administration in such patients.

## 1. Introduction

Thrombotic thrombocytopenic purpura (TTP) is a potentially life-threatening consumptive coagulopathy requiring emergent diagnosis and timely treatment. It is characterized by microangiopathic hemolytic anemia and thrombocytopenia with the development of microthrombi caused by severely reduced activity of the von Willebrand factor-cleaving protease ADAMTS13 and resulting end-organ damage. The central nervous system is commonly affected and may manifest with mental status or functional neurologic changes. Renal involvement may lead to cardiac manifestations associated with volume overload or arrhythmogenic electrolyte abnormalities. TTP is a result of an inherited or acquired quantitative or functional deficiency of ADAMTS13. Our case report features a patient with a history of idiopathic thrombocytopenic purpura and thrombotic thrombocytopenic purpura, who developed a recurrence of TTP 5 days after his first dose of the Moderna mRNA COVID-19 vaccine (mRNA-1273 vaccine). The FDA issued an Emergency Use Authorization for the Moderna mRNA COVID-19 vaccine on December 18, 2020. It is a novel vaccine type that has demonstrated 94.1% efficacy at preventing COVID-19 in an ongoing phase 3 clinical trial. Vaccine distribution to a more extensive and more diverse population will allow for an increased rate of adverse event reporting.

## 2. Case Description

The patient is a 48-year-old Caucasian male who presented complaining of acute-onset, transient right-sided weakness and slurred speech lasting approximately 30 minutes in addition to paresthesia five days after his first dose of COVID-19 mRNA vaccine (Moderna mRNA-1273 vaccine). The patient did not have headache, dyspnea, fever, or visual changes. Vitals were within the normal range. The patient had a normal cardiopulmonary exam, and his neurological exam was intact with complete recovery of transient symptoms. Examination of the skin and mucosa did not show any petechiae, purpura, or active bleeding. He denied any recent medication use, illness, or chemical exposure. The patient's past medical history was significant for diabetes mellitus type 2 on insulin, refractory thrombotic thrombocytopenic purpura (TTP), idiopathic thrombocytopenic purpura (ITP), and COVID-19 2 months before his presentation, which did not require hospitalization. His medical records elucidated that he had a diagnosis of TTP for eight years, a total of 4 episodes in the past for which he was treated with plasmapheresis, steroids, and rituximab. Further treatment options were not initiated since the patient was lost to follow-up in between his relapses. He was also diagnosed with ITP 5 years ago, which was treated with steroids and rituximab. The patient was reported to have normal platelet counts (130.000/uL) approximately three months before the presentation. Family history was nonpertinent. He denied consumption of alcohol, tobacco, or recreational drugs. The patient denied taking any home medications.

On the day of presentation, laboratory results were significant for hemolytic anemia with hemoglobin 8.8 g/dL, 2–3 schistocytes/hpf on peripheral blood smear, haptoglobin<10 mg/dL, total bilirubin 5.0 with indirect bilirubin predominance, lactate dehydrogenase (LDH) 884 U/L, D-dimer 1.45 ug/mFEU, fibrinogen 417 mg/dL, and platelet count 10 × 103/dL (baseline platelet count of 130 × 103/dL). Noncontrast head CT was performed given acute transient neurologic symptoms which did not reveal any hemorrhage, mass effect, or ischemic process. The constellation of hemolytic anemia, thrombocytopenia, and transient neurological symptoms was concerning for a TTP relapse. The PLASMIC score used to predict ADAMTS13 deficiency in suspected TTP patients was 7 points showing that the patient is at high risk of having severe ADAMTS13 deficiency, thus requiring emergent plasmapheresis. ADAMTS13 activity later resulted as <3%, while ADAMTS13 inhibitor was 6.6 BEU (normal <0.4). Meanwhile, the patient's COVID-19 PCR test was negative. High-dose glucocorticoids were immediately administered, and fresh frozen plasma was used for bridging to urgent plasmapheresis. The patient had a full recovery after a 10-day course of plasmapheresis in addition to glucocorticoids and rituximab. The patient was to complete outpatient rituximab therapy and steroid tapering with hematology follow-up on discharge. His platelet and hemoglobin count was normalized on the day of discharge. The second dose of the COVID-19 vaccine was deferred.

## 3. Discussion

TTP is a result of an inherited or acquired quantitative or functional deficiency of ADAMTS13. Acquired TTP is characterized by severe ADAMTS13 deficiency (typically, activity <10 percent) due to an inhibitor (autoantibody) directed against ADAMTS13; patients with inherited ADAMTS13 mutations are referred to as having hereditary TTP. Reduction in ADAMTS13 activity leads to the accumulation of von Willebrand factor multimers followed by an increase in platelet aggregation and thrombotic microangiopathy. The disease manifests with thrombocytopenia, mechanical hemolysis, anemia, renal failure, neurologic impairment, and fever. Acquired TTP diagnosis is confirmed when ADAMTS13 activity is <10%. ADAMTS13 assay typically requires days and, thus, is not an actionable measure and instead acts as a confirmatory test. Acute diagnosis relies on clinical presentation and laboratory data. Laboratory data demonstrate thrombocytopenia, microangiopathic hemolytic anemia (anemia in conjunction with lab values of cell lysis: elevated LDH, decreased haptoglobin, and indirect hyperbilirubinemia), reticulocytosis, and peripheral smear with schistocytes. The platelet-rich microvascular thrombi lead to organ hypoperfusion. In our case, as we had a high suspicion for TTP initially given the past medical history of TTP, hemolytic anemia with thrombocytopenia with neurologic symptoms, we initiated plasmapheresis without waiting ADAMTS13 levels. We confirmed the diagnosis with low ADAMTS13 levels which were withdrawn before plasmapheresis. MRI of the brain was not obtained as the neurologic symptoms were transient and resolved entirely once the underlying etiology improved. Of note, per documentation, our patient had past medical history of ITP, which was not deemed as the etiology of his presentation at this time given hallmarks of thrombotic microangiopathic anemia and low levels of ADAMTS13 levels. Concomitant ITP with TTP is a rare phenomenon, per the patient's external medical records, and he had ITP in the past; however, we did not have adequate data to confirm.

The timing of TTP identification and treatment determines disease mortality. The PLASMIC score predicts disease severity and potential response to plasmapheresis [1]. Treatment primarily involves plasmapheresis, steroids, and rituximab. Plasmapheresis removes circulating autoantibodies while also supplying functionally active ADAMTS13. Steroids suppress the immune system. Relapsing TTP is defined as thrombocytopenia and increased LDH that persists despite 5–7 plasmapheresis sessions [2]. Rituximab, a monoclonal anti-CD20 antibody, results in B-cell depletion and has been shown to reduce the rate of TTP recurrence. Caplacizumab is an anti-VWF antibody fragment that has been shown to normalize platelet count faster with shorter hospitalization, fewer plasmapheresis sessions, and lower rates of composite death, recurrence, and major thromboembolic events compared with placebo [2].

Although the exact etiology is unknown, acquired TTP can be triggered by bacterial and viral infections, pregnancy, malignancy, and medications (including quinine, cyclosporine, and mitomycin C). In the literature, only few publications reported vaccination-induced TTP [3–8]. Vaccinations can rarely induce autoimmune reactions including acquired TTP. According to these case reports, TTP occurred within 2 weeks after vaccination, mostly with influenza vaccination (*n* = 3), followed by vaccines against pneumococcus (*n* = 1), rabies (*n* = 1), and H1N1 (*n* = 1). To date, COVID-19 vaccination associated is present in the literature. Literature is evolving for COVID-19 coagulopathy; although the mechanism is still unclear, COVID-19 promotes a hypercoagulable state with wide-spectrum clinical manifestations. According to a recent literature review, four cases of TTP related to COVID-19 were described [9]. Three of the four patients had plasmapheresis with steroids. However, one of them could not have plasmapheresis due to logistics but responded well to fresh frozen plasma and IVIG therapy. Two of the cases required rituximab therapy as full recovery was not achieved with plasmapheresis. The literature review demonstrated two TTP cases related to mRNA COVID-19 vaccine administration without any hematologic disorders and a possible trigger for TTP occurrence. A case of TTP with a 62-year-old female without any hematologic disease was identified after 37 days of Ad26.COV2-S administration was seen, treated with plasmapheresis and high-dose steroids, and the outcome was not reported. Another case was a 38-year-old woman with a de novo iTTP, two weeks after exposure to BNT-162b2, who was successfully treated with plasma exchange, corticosteroids, rituximab, and caplacizumab.

Our patient's COVID-19 test resulted positive via nasopharyngeal swab PCR, two months before COVID-19 vaccination. At the time of the COVID-19 infection, he was asymptomatic and quarantined for 14 days. In the COVID mRNA-1273 trial [10], 2.3% in the mRNA-1273 group demonstrated serologic and/or virologic evidence of SARS-CoV-2 infection prior to vaccination. The primary endpoint of the study was to determine efficacy in preventing symptomatic COVID-19 in those without prior infection. Although neutralizing antibody titers (SARS-CoV-2 spike IgG) have been measured after vaccination to assess appropriate response, B and T (CD4+ and CD8+) memory cells may be more important in those with previous infection [11, 12]. Participants with prior infection who received mRNA vaccine tended to have a more robust and uniform antibody response to the first dose [13]. In contrast, there was no increase in antibody response to the second dose. The intensity of antibody production in previously infected individuals seems to reflect heightened reactogenicity to the mRNA vaccine. Reactogenicity was higher in those with preexisting immunity, as systemic side effects (fatigue, headache, chills, muscle pain, fever, and joint pain) occurred more frequently [13]. In TTP, this level of immune system stimulation may have led to anti-ADAMTS13 autoantibody production. The interval between infection and vaccination was approximately two months in this case. Most studies have started vaccine administration 30 to 60 days after seropositive status. Measuring antibody titer before vaccination may help inform vaccine schedule, as immunogenic potential may depend on this. However, immunologic response to infection may vary based on viral load and other individual factors and, thus, is unpredictable. Our patient exemplifies recurrence of TTP after COVID-19 mRNA vaccination in the setting of recent COVID-19. Given the lack of any other possible trigger, including sepsis, HIV, malignancy, and medications, we pose that COVID-19 vaccination shortly after COVID-19 caused a relapse of TTP. Considering the patient's COVID-19 two months before vaccination, enhanced immune response after the vaccination might have resulted in a relapse of the underlying autoimmune disorder.

Widespread vaccination against COVID-19 will be playing a key role in fighting the ongoing pandemic. Meanwhile, vaccine administration to a more extensive and more diverse population will allow for adverse event reporting. This case report exemplifies potential safety issues encountered with new vaccine administration in patients, especially shortly after COVID-19 with underlying autoimmune disease.

## Data Availability

Data can be provided upon request by the responding author.

## References

[1] Bendapudi P. K., Hurwitz S., Fry A. (2017). Derivation and external validation of the PLASMIC score for rapid assessment of adults with thrombotic microangiopathies: a cohort study. *The Lancet Haematology*.

[2] Kalpatthi R., Kiss J. E. (2020). Thrombotic thrombocytopenic purpura, heparin-induced thrombocytopenia, and disseminated intravascular coagulation. *Critical Care Clinics*.

[3] Kadikoylu G., Yavasoglu I., Bolaman Z. (2014). Rabies vaccine-associated thrombotic thrombocytopenic purpura. *Transfusion Medicine*.

[4] Kojima Y., Ohashi H., Nakamura T. (2014). Acute thrombotic thrombocytopenic purpura after pneumococcal vaccination. *Blood Coagulation and Fibrinolysis*.

[5] Ramakrishnan N., Parker L. P. (1998). Thrombotic thrombocytopenic purpura following influenza vaccination—a brief case report. *Connecticut Medicine*.

[6] Dias P. J., Gopal S. (2009). Refractory thrombotic thrombocytopenic purpura following influenza vaccination. *Anaesthesia*.

[7] Hermann R., Pfeil A., Busch M. (2010). Schwerste thrombotisch-thrombozytopenische Purpura (TTP) nach H1N1-Vakzinierung. *Medizinische Klinik*.

[8] Brown R. C., Blecher T. E., French E. A., Toghill P. J. (1973). Thrombotic thrombocytopenic purpura after influenza vaccination. *BMJ*.

[9] Tehrani H. A., Darnahal M., Vaezi M., Haghighi S. (2021). COVID-19 associated thrombotic thrombocytopenic purpura (TTP); A case series and mini-review. *International Immunopharmacology*.

[10] Baden L. R., El Sahly H. M., Essink B. (2021). Efficacy and safety of the mRNA-1273 SARS-CoV-2 vaccine. *New England Journal of Medicine*.

[11] Sette A., Crotty S. (2021). Adaptive immunity to SARS-CoV-2 and COVID-19. *Cell*.

[12] Krammer F., Srivastava K., Alshammary H. (2021). Antibody responses in seropositive persons after a single dose of SARS-CoV-2 mRNA vaccine. *New England Journal of Medicine*.

[13] Widge A. T., Rouphael N. G., Jackson L. A. (2021). Durability of responses after SARS-CoV-2 mRNA-1273 vaccination. *New England Journal of Medicine*.

